# Ethnicity-stratified analysis of the association between XRCC3 Thr241Met polymorphism and leukemia: an updated meta-analysis

**DOI:** 10.1186/s12920-021-01076-w

**Published:** 2021-09-18

**Authors:** Zhengjun Xie, Wei Peng, Qiuhua Li, Wei Cheng, Xin Zhao

**Affiliations:** Department of Hematology, The Fifth Affiliated Hospital of Zunyi Medical University, Zhufeng Avenue 1439, Zhuhai, 519000 China

**Keywords:** Leukemia, Genetic polymorphism, XRCC3, Meta-analysis

## Abstract

**Background:**

Presently, whether X-ray repair cross complementing group 3 (XRCC3) Thr241Met polymorphism is correlated to leukemia risk remains controversial. Because of this reason, the objective of current study is to explore whether XRCC3 Thr241Met polymorphism confers risk to leukemia.

**Methods:**

Two independent authors systematically and comprehensively searched Pubmed, Embase, the Cochrane library, Google academic, China National Knowledge Infrastructure (CNKI). Search time is from database foundation to March 2021.

**Results:**

Overall, significant associations between leukemia risk and XRCC3 Thr241Met polymorphism were found in Caucasian population by allele contrast (T vs. C: OR 1.20, 95% CI 1.02–1.40), homozygote comparison (TT vs. CC: OR 1.35, 95% CI 1.05–1.73), and recessive genetic model (TT vs. TC/CC: OR 1.31, 95% CI 1.04–1.64).

**Conclusions:**

The present meta-analysis suggests that the XRCC3 Thr241Met polymorphism may be a risk factor for leukemia in Caucasian population.

**Supplementary Information:**

The online version contains supplementary material available at 10.1186/s12920-021-01076-w.

## Background

Leukemia is a very frequent malignance tumor originating from hematopoietic stem cells. The leukemia cell stops at different stages of cell development due to uncontrolled proliferation, dysdifferentiation and aleukemiatosis block. Its common symptoms are anemia, infection and bleeding. The incidence of leukemia in China is approximately 3–8 individuals per 100,000 [1, 2]. About One hundred thousand people are diagnosed with leukemia every year [2]. There is no doubt that the occurrence of leukemia brings a huge burden on individuals, families, and health care systems. However, its exact etiology and pathogenesis remains unknown.

Several studies have shown that the occurrence of leukemia is associated with exposure to risk environment factors such as benzene, formaldehyde, smoking history, residence decoration and the use of different kinds of hair dye. Benzene and its metabolites make bone marrow damaged by immune-mediated responses, leading to the occurrence of leukemia [3]. A large-scale cohort study has shown that exposure to environmental benzene is associated with a variety of hematological malignancies, including acute leukemia, MDS, and T-cell lymphoma [4]. With the increased dose and frequency of hair colorants, the micronucleus rate of polychromatic erythrocytes (PCE) in bone marrow of mice increased, suggesting hair colorants can cause chromosomal damage, and long-term use of hair colorants will increase the risk of acute leukemia. Indoor decoration materials can release hundreds of pollutants such as benzene, formaldehyde, radon and volatile harmful gases. A number of epidemiological studies have shown that short occupancy time after decoration is associated with the incidence of leukemia [5].

The occurrence and development of leukemia is a complicated process. Many scholars attribute it to some risk factors including physical factors, chemical factors, and virus infection; however, these factors are not acting as a necessary role for leukemia occurrence and progression. Approximate 30% patients do not embrace physical factors, chemical factors, and virus infection will acquire leukemia. All the above evidences indicate that extra genetic or non-genetic factors modulating leukemia susceptibility are yet to be identified.

Although leukemia pathogenesis is an extremely complicated process and the exact pathogenesis of leukemia is still unknown, studies have shown that DNA damage is closely related to its occurrence and development [6]. Many researchers have shown that multiple forms of DNA damage can occur and double-stranded DNA breakage is the most common type, leading to cell death, loss of genetic material, and translocation or deletion of chromosomes. On the other hand, there are many complex mechanisms in the body to maintain the stability of genetic material, including DNA repair pathways, antioxidant stress systems and anti-damage factor systems [7]. Double-stranded fracture repair is a form of DNA repair pathway, which also includes homologous recombination repair and non-homologous recombination repair [8]. XRCC3 is an important protein during the process of DNA homologous recombination repair, and its single nucleotide polymorphisms play an important role on DNA homologous recombination repair [9, 10]. Some studies have suggested that XRCC3 Thr241Met polymorphism is associated with leukemia risk. But other studies hold the controversial idea.

Yan et al. published a literature in 2014 that also investigates the association between XRCC3 Thr241Met polymorphism and leukemia risk [11]. Regrettably, only seven studies were included in their meta-analysis. They concluded that XRCC3 Thr241Met polymorphism was not associated with leukemia risk. Qin et al. published a literature in 2013 that also investigates the association between XRCC3 Thr241Met polymorphism and leukemia risk [12]. Similarly, they also get a negative result. Compared with the previous meta-analysis, some important advantages of our paper should be pointed out. Firstly, more eligible studies were enrolled in our meta-analysis. By this means, 16 literatures (10 Caucasian, 4 Asian, and 2 African) were included. Compared with previous meta-analysis, the number of eligible literatures greatly increased. And the merit of meta-analysis is just improving statistical efficiency and making the results more truthful. What’s more, the present study reverses the previous results. We have first discovered that XRCC3 Thr241Met polymorphism contributes an increased risk to leukemia of Caucasian population. The results of our study indicate the limited sample size of previous meta-analysis. So that we think the present meta-analysis is reliable and comprehensive.

As far as we know, this is the first meta-analysis which comprehensively explores the association between XRCC3 Thr241Met polymorphism and leukemia susceptibility. The objective of current study is to estimate whether XRCC3 Thr241Met polymorphism confers risk to leukemia.

## Materials and methods

### Search strategy

Two independent authors systematically and comprehensively searched Pubmed (https://www.ncbi.nlm.nih.gov/pubmed/), EMBASE (https://www.embase.com/), the Cochrane library(https://www.cochranelibrary.com/), Google academic (https://scholar.google.com/), and Chinese national knowledge internet (https:// www.cnki.net/). Search time is from database foundation to March 2021. The keywords applied in the search process were as follows: (“XRCC3” or “X-ray repair cross complementing group 3”) together with (“leukemia”). The literature language was limited to English language and Chinese language. Additionally, in order to avoid the omission of relevant literatures, we searched the references as much as possible.

### Inclusion and exclusion criteria

The inclusion criteria must meet a series of conditions: (a) a case–control study; (b) making an assessment of the association between XRCC3 Thr241Met polymorphism and leukemia risk; (c) offering sufficient information and data to count OR and 95%CI. The exclusion criteria also must meet a few conditions: (a) patients with other hematological system diseases such as multiple myeloma, aplastic anemia, myelodysplastic syndrome, autoimmune hemolytic anemia, idiopathic thrombocytopenic purpura. (b) patients with some inflammatory diseases or cardiovascular and cerebrovascular diseases such as urinary tract infection or shock, acute myocardial infarction or unstable angina, rheumatoid arthritis or systemic lupus erythematosus. (c) not offering sufficient data for meta-analysis. (d) its experiment objective was pig, rat or other animals.

### Data extraction and methodological quality assessment

All the necessary information was independently reviewed and assessed by first author and second author (Zhengjun Xie; Wei Peng). Then this contradictory data or information was reassessed by the third author (Qiuhua Li). The extracted data consisted of author name, publication year, genotyping methods, sample size, ethnicity, matching criteria, source of control, HWE conformity. If the similar opinion could not reach in the course of data extraction, suggestion was offered by another experienced researcher (Qiuhua Li) to determine the correct selection. The similar method was applied equally to evaluation of literature quality. In the present meta-analysis, we applied the risk assessment criteria of Newcastle–Ottawa Scale (NOS) bias to evaluate the quality of each literature. The main criteria consisted of three aspects including selection of enrolled study subjects (0–4 scores); between-group comparability (0–2 scores); exposure outcomes and factors (0–3 scores). It should be noted that the ethics approval of our study was waived by Ethics Committee of The Fifth Affiliated Hospital of Zunyi Medical University as no human or animal was directly enrolled in our study and meta-analysis is the statistical analysis of large collection of analysis results from individual studies for the purpose of integrating the findings.

### Statistical analysis

The association power was assessed through the corresponding indexes including OR and 95%CI. And both the Q-statistic and I^2^ statistics would be applied [13]. Four genetic models were applied in the present meta-analysis including allele contrast (T vs. C), homozygote comparison (TT vs. CC), heterozygous comparison (TC vs. CC), recessive genetic model (TT vs. TC/CC) and dominate genetic model (TT/TC vs. CC). The model of fixed-effects and random-effects would be put into use on the basis of heterogeneity degree [14, 15]. I^2^ < 50% was considered to low heterogeneity, 50 ≤ I^2^ < 75% was considered to moderate heterogeneity and I^2^ ≥ 75%was considered to significant heterogeneity. If I^2^ < 50% and P > 0.1, the fixed-effects model would be used. If I^2^ ≥ 50% or P ≤ 0.1, the random -effects model would be used. Furthermore, the Galbraith plot was used to spot the outliers to find out the potential heterogeneity as much as possible. Sensitive analysis was applied to detect the influential studies which might contribute obvious bias to final results. The funnel plot and Egger’s test were put into use to recognize the existence of publication bias [16]. Meta-regression and subgroup analysis were used to detect and deal with the possible source of heterogeneity. The Stata 12.0 would be responsible for the whole statistics. The meta-analysis was conducted based on the Preferred Reporting Items for Systematic Reviews and Meta-Analyses (PRISMA) 2009 checklist (Additional file 1: Table S1 Checklist) [17, 18]. Furthermore, HWE conformity was based on the P value of control group (P > 0.05 was considered HWE conformity).

## Results

### General information

PRISMA 2009 Flow Diagram shows the flow chart of meta-analysis search course (Additional file 2: Table S2 Checklist) [17, 18]. Based on the search strategy, sixteen literatures were satisfactory [19–34]. Table 1 shows the detailed information of all literatures. In total, sixteen literatures consisted of ten literatures from European countries and America, four literatures from Asian countries and two literatures from African countries. Different genotyping methods were used such as direct sequencing, PCR–RFLP and TaqMan. The publication year ranged from 2002 to 2018 and the controls were population-based or hospital-based. All the genotyping frequency of controls was conform to HWE. And the sample size ranged from 80 to 1600.

**Table 1:** Main characteristics of all case–control studies included in meta-analysis

Literature	Ethnics (country)	Genotyping methods	Source of control	Sample size	HWE conformity	NOS	Genotype frequency (Case)			Genotype frequency (Control)			Mean age (Case)	Mean age (Control)	Year
							CC	CT	TT	CC	CT	TT			
Seedhouse et al*.* [15]	Caucasian (United Kingdom)	PCR–RFLP	PB	123/175	Yes	8	99	87	30	92	64	19	63 (17–96)	52 (15–97)	2002
Seedhouse et al*.* [16]	Caucasian (United Kingdom)	PCR–RFLP	PB	216/175	Yes	8	119	103	38	92	64	19	64 (11–96)	50 (15–97)	2004
Matullo et al*.* [17]	Caucasian (European countries)	TaqMan	PB	169/1094	Yes	8	61	90	18	383	544	167	35–74	35–74	2006
Bhatla et al*.* [18]	Caucasian (United States)	TaqMan	PB	282/646	Yes	9	125	157	47	253	309	84	NR	NR	2008
Zhang et al*.* [19]	Asian (China)	PCR–RFLP	PB	148/458	Yes	7	133	13	2	403	46	9	42 (23–76)	42 (23–76)	2009
Hamdy et al*.* [20]	African (Egypt)	Direct sequencing	PB	50/30	Yes	6	22	20	8	18	9	3	14–65	12–46	2011
Liu et al*.* [21]	Asian (China)	PCR–RFLP	PB	379/806	Yes	7	55	39	11	627	73	4	32 (5–69)	42 (15–90)	2011
Nina et al*.* [23]	Caucasian (Slovenia)	TaqMan	PB	20/39	Yes	7	6	8	6	15	19	5	9.5 (2–34)	10 (0–37)	2012
Abramenko et al*.* [22]	Caucasian (Ukraine)	PCR–RFLP	PB	159/73	Yes	8	74	60	25	30	33	10	57.78 ± 1.09	58.16 ± 0.91	2012
Sorour et al*.* [25]	African (Egypt)	PCR–RFLP	PB	90/60	Yes	7	24	63	3	12	42	6	16–60	18–69	2013
Banescu et al*.* [24]	Caucasian (Romania)	PCR–RFLP	PB	78/121	Yes	7	36	30	12	85	27	9	51.76 ± 17.1	58.84 ± 12.9	2013
Smolkova et al*.* [27]	Caucasian (Germany)	TaqMan	PB	459/549	Yes	9	178	216	65	216	256	77	6.9 ± .4.4	32 ± 8.1	2014
Banescu et al*.* [26]	Caucasian (Romania)	PCR–RFLP	PB	78/121	Yes	7	64	70	22	85	79	16	51.5 ± 1.1	49.8 ± 2.1	2014
Miao et al*.* [28]	Asian (China)	TaqMan	PB	545/1034	Yes	9	470	45	3	902	130	1	46 (8–80)	43 (8–85)	2015
Mutlu et al*.* [29]	Caucasian (Turkey)	PCR–RFLP	HB	25/30	Yes	7	9	12	4	13	11	6	NR	NR	2015
Pei et al*.* [30]	Asian (Taiwan)	PCR–RFLP	PB	266/266	Yes	7	214	39	13	241	19	6	7.0 ± 4.4	8.3 ± 4.8	2018

*PB* population-based, *HWE* Hardy–Weinberg equilibrium, *RFLP* restricted fragment length polymorphism, *NOS* Newcastle–Ottawa Score, *NR* not reported

### Meta-analysis results

**Fig. 1:**
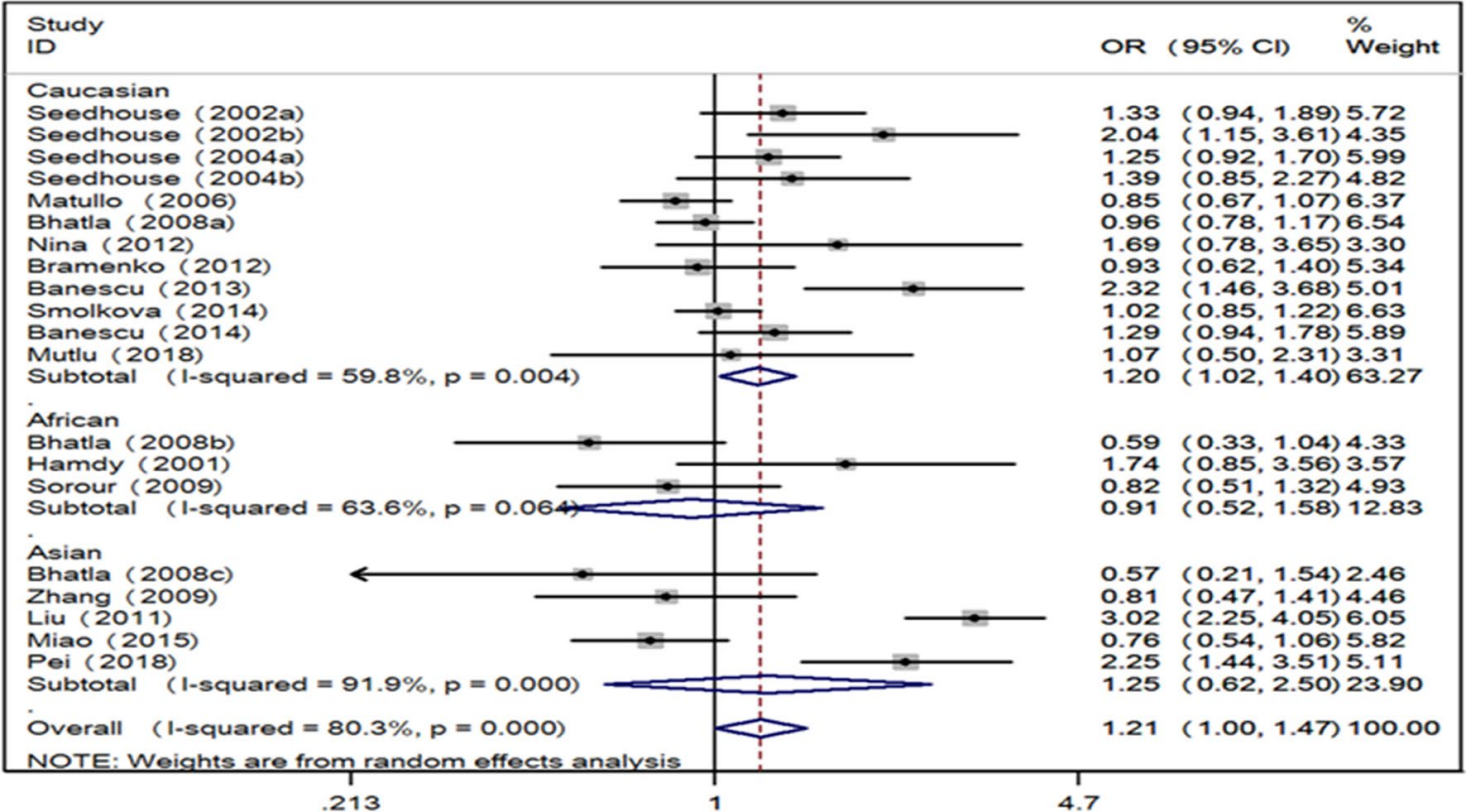
Forest plot for the associations between XRCC3 Thr241Met polymorphism and leukemia risk through allele contrast (T vs. C). *XRCC3* X-ray repair cross complementing group 3, *OR* odds ratio, *CI* confidence interval

**Fig. 2:**
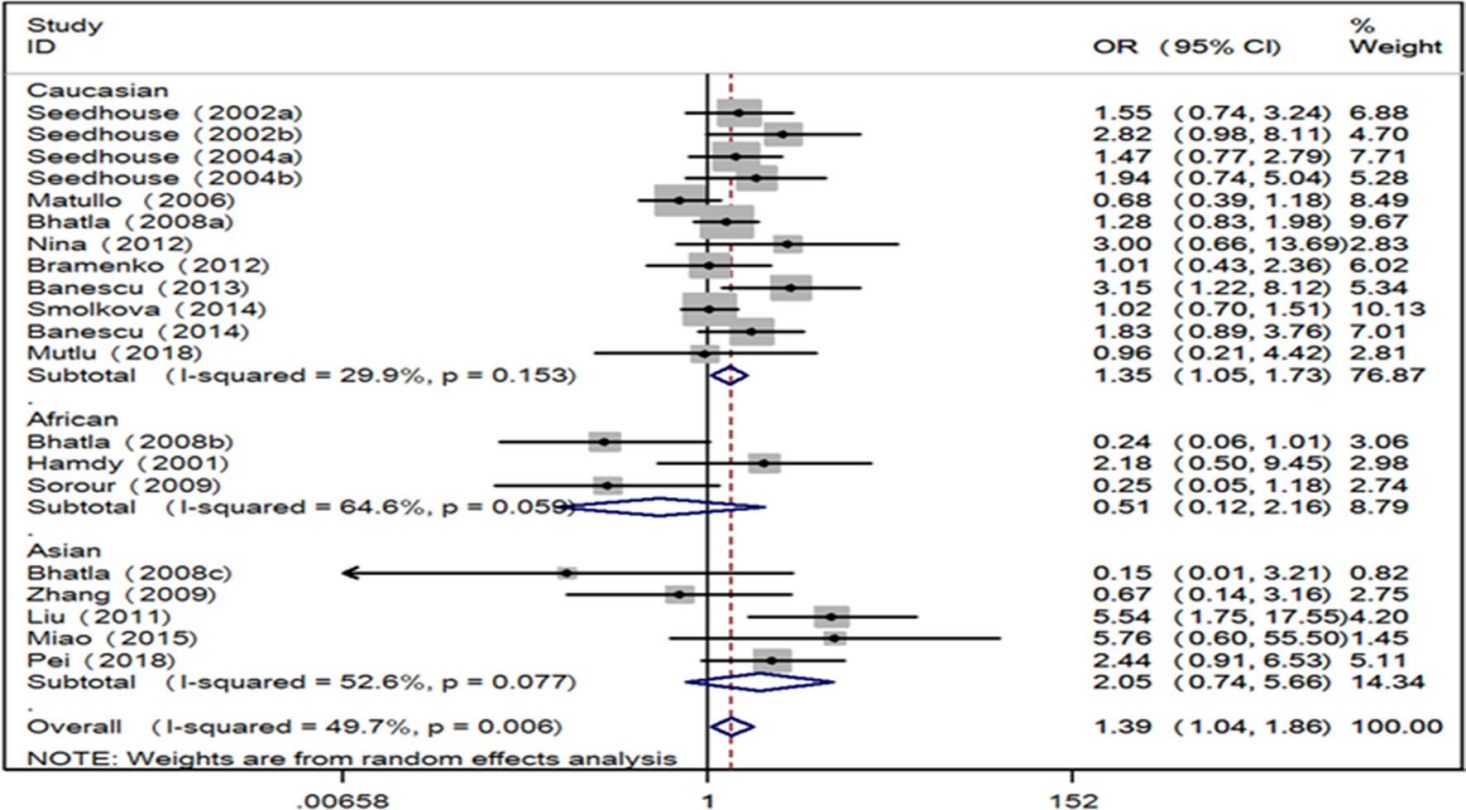
Forest plot for the associations between XRCC3 Thr241Met polymorphism and leukemia risk through homozygote comparison (TT vs. CC). XRCC3, X-ray repair cross complementing group 3; *OR* odds ratio, *CI* confidence interval

**Fig. 3:**
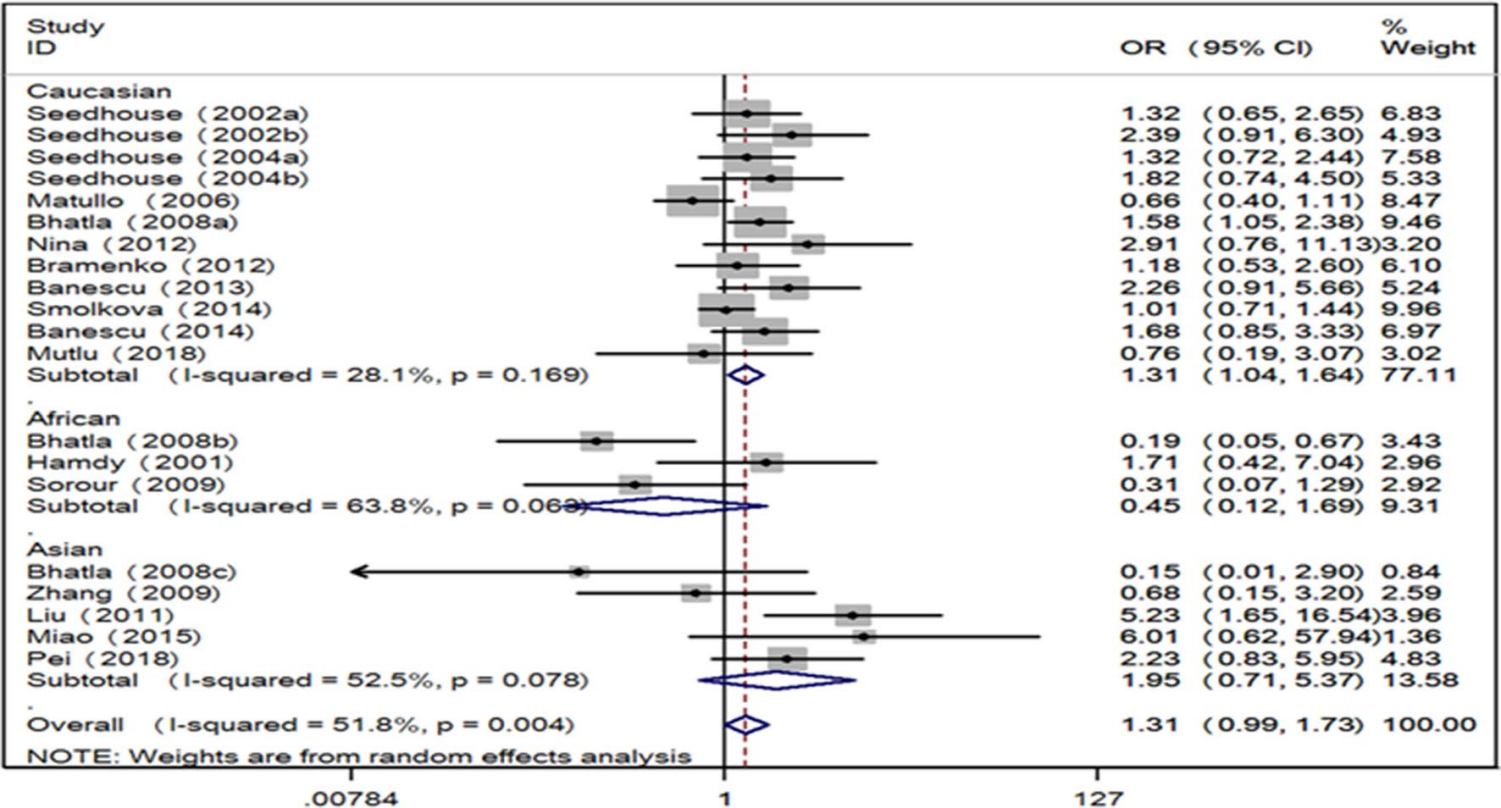
Forest plot for the associations between XRCC3 Thr241Met polymorphism and leukemia risk through recessive genetic model (TT vs. TC/CC). *XRCC3* X-ray repair cross complementing group 3, *OR* odds ratio, *CI* confidence interval

The meta-analysis results between XRCC3 Thr241Met polymorphism and leukemia susceptibility are shown in Table 2. Generally, positive finding between leukemia and XRCC3 Thr241Met polymorphism was found in Caucasian population by allele contrast (T vs. C: OR 1.20, 95% CI 1.02–1.40, P = 0.026, Fig. 1), homozygote comparison (TT vs. CC: OR 1.35, 95% CI 1.05–1.73, P = 0.018, Fig. 2), and recessive genetic model (TT vs. TC/CC: OR 1.31, 95% CI 1.04–1.64, P = 0.023, Fig. 3).

**Table 2:** The general results for the association between XRCC3 Thr241Met polymorphism with leukemia risk

Comparison	Group	N	Test of association			Mode	Test of heterogeneity		
			OR	95% CI	P		χ^2^	P	I^2^
T versus. C	Overall	20	1.21	1.00–1.47	0.049	Random	96.21	0	80.3
	Caucasian	12	1.20	1.02–1.40	**0.026**	Random	27.38	0.004	59.8
	Asian	5	1.25	0.62–2.50	0.530	Random	49.34	0	91.9
	African	3	0.91	0.52–1.58	0.727	Random	5.50	0.064	63.6
TT versus. CC	Overall	20	1.39	1.04–1.86	0.027	Random	37.78	0.006	49.7
	Caucasian	12	1.35	1.05–1.73	**0.018**	Fixed	15.69	0.153	29.9
	Asian	5	2.05	0.74–2.66	0.169	Random	8.44	0.077	52.6
	African	3	0.51	0.12–2.16	0.361	Random	5.65	0	64.6
TC versus. CC	Overall	20	1.05	0.84–1.34	0.443	Random	32.99	0.005	55.8
	Caucasian	12	1.07	0.86–1.32	0.123	Fixed	11.22	0.166	30.4
	Asian	5	1.01	0.54–1.76	0.643	Random	9.62	0.078	54.8
	African	3	1.25	0.77–1.98	0.255	Random	7.32	0.002	62.1
TT versus. TC + CC	Overall	20	1.31	0.99–1.73	0.063	Random	39.41	0.004	51.8
	Caucasian	12	1.31	1.04–1.64	**0.023**	Fixed	15.30	0.169	28.1
	Asian	5	1.95	0.71–5.37	0.194	Random	8.42	0.078	52.5
	African	3	0.45	0.12–1.69	0.239	Random	5.52	0.063	63.8
TT + TC versus. CC	Overall	20	1.19	0.99–1.43	0.071	Random	49.23	0	61.4
	Caucasian	12	1.18	0.97–1.44	0.104	Random	23.28	0.016	52.8
	Asian	5	1.19	0.69–2.05	0.537	Random	22.31	0	82.1
	African	3	1.03	0.57–1.87	0.928	Random	2.81	0.246	28.8

Bold values emphasize P < 0.05

*OR* odds ratio, *95% CI* 95% confidence interval

### Evaluation of heterogeneity and sensitivity

**Fig. 4:**
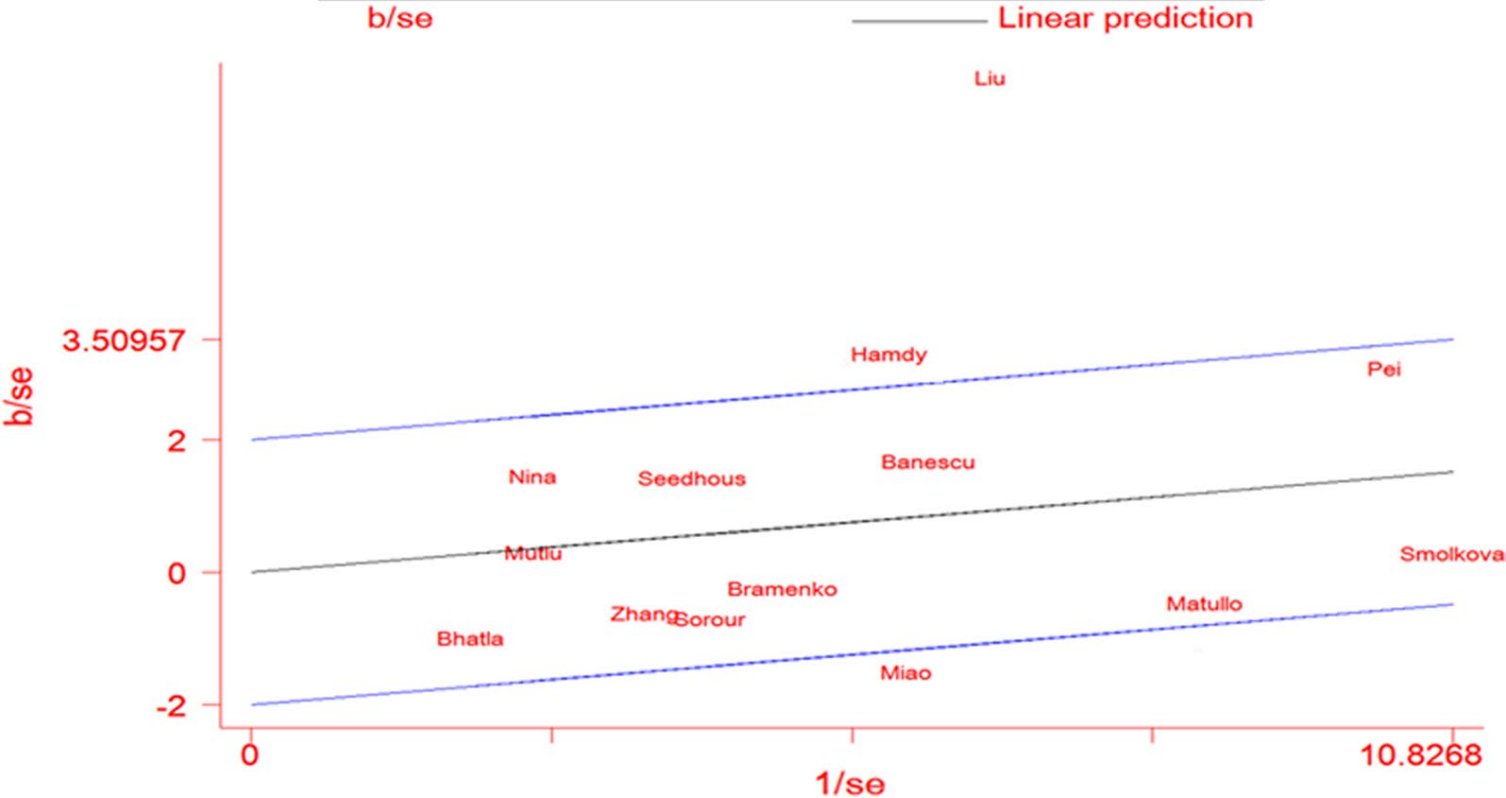
Galbraith plot of XRCC3 Thr241Met polymorphism and leukemia risk by allele contrast: A vs. G. *XRCC3* X-ray repair cross complementing group 3

Significant heterogeneity was found under all the allele contrast (χ^2^ = 141.02, P = 0, I^2^ = 86.5, Table 2), homozygote comparison (χ^2^ = 59.73, P = 0, I^2^ = 68.2, Table 2), recessive genetic model (χ^2^ = 55.58, P = 0, I^2^ = 65.8, Table 2), and dominate genetic model (χ^2^ = 109.36, P = 0, I^2^ = 82.6, Table 2). To detect the possible source of heterogeneity, we conducted meta-regression and subgroup analysis. Meta-regression revealed that ethnicity was the main source of heterogeneity which contributed substantial heterogeneity to the final results. Then we conduct subgroup analyses stratified by ethnicity. Subsequently, the heterogeneity reduced in Caucasian population under allele contrast (χ^2^ = 27.38, P = 0.004, I^2^ = 59.8, Table 2), homozygote comparison (χ^2^ = 15.69, P = 0.153, I^2^ = 29.9, Table 2), recessive genetic model (χ^2^ = 15.30, P = 0.169, I^2^ = 28.1, Table 2), and dominate genetic model (χ^2^ = 23.28, P = 0.016, I^2^ = 52.8, Table 2). In order to further detect the source of heterogeneity of African and Asian population, we conduct Galbraith plots to find out the outliers which might influence the heterogeneity. Consequently, we found the studies Liu et al. and Hamdy et al. were not within reasonable limits (Fig. 4). Then we excluded two studies and performed meta-analysis again, we found that the results were not altered.

#### Sensitivity analysis and Publication Bias

**Fig. 5:**
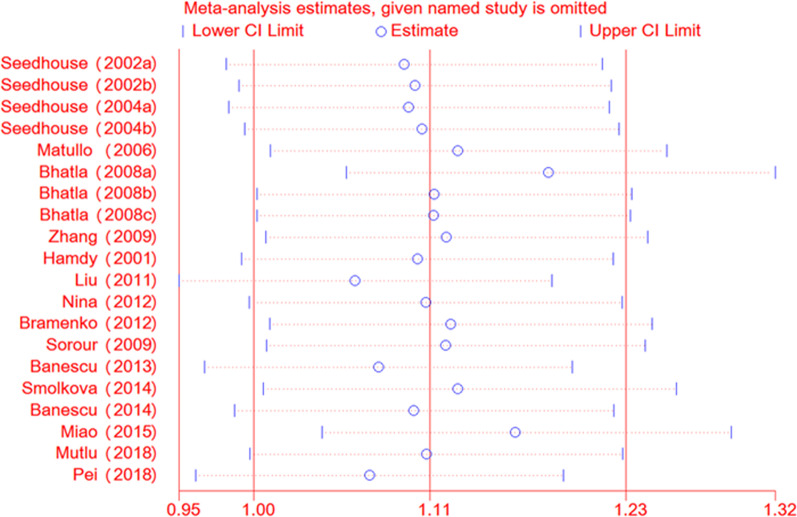
Sensitive analysis of XRCC3 Thr241Met polymorphism and leukemia risk by allele contrast: A vs. G. *XRCC3* X-ray repair cross complementing group 3

**Fig. 6:**
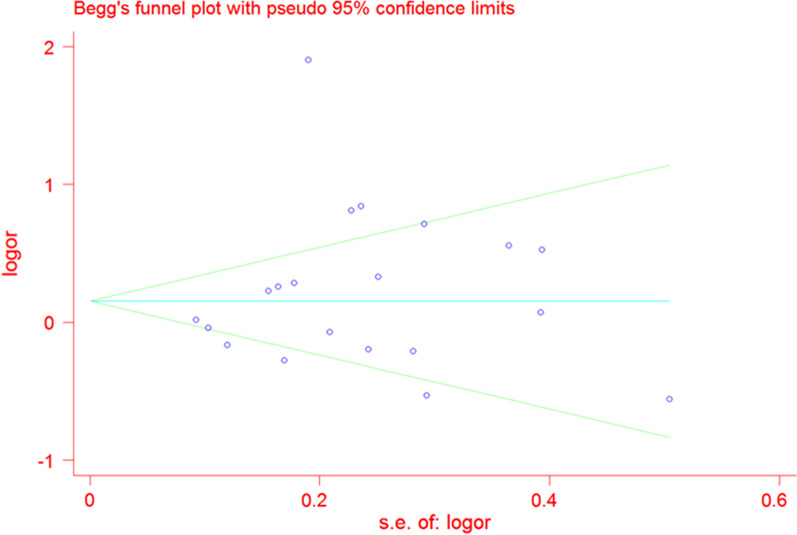
Funnel plot on publication bias for the associations between XRCC3 Thr241Met polymorphism and susceptibility to leukemia through the allele model (T vs. C). *XRCC3* X-ray repair cross complementing group 3, *SE* standard error, *OR* odds ratio

To verify the reliability and stability of meta-analysis results, sensitive analysis was applied to detect the influential studies which might contribute obvious bias to final results. The final results were not altered by any single literature, suggesting that the results of our meta-analysis were stable and reliable (Fig. 5).We only find mild asymmetrical by funnel plot (P = 0.881) (Fig. 6 and Additional file 3: Figure S3). And we do not find any evident publication bias by Egger’s test in any genetic model (P = 0.486, 0.682, 0.514, 0.407, 0.357, respectively).

## Discussion

Considering the increasing prevalence of leukemia and its percentage among population death causes, leukemia prevention and treatment are always one of the key medical research subjects in all countries. The morbidity is the highest in some developing countries including China, Iran, Thailand, Pakistan, Mexico and Latin America, and the morbidity can reach 2.5–8 individuals per 100,000 [35–42]. The leukemia not only poses a threat to People’s health and lives but also brings huge economic burden and mental pressure to the society and families. Nevertheless, it is well-established that the etiology of leukemia is awfully complicated and the role of etiology remains to be elucidated.

Although the pathogenesis of leukemia is a complex process, one thing for sure is that its pathogenesis is mainly caused by the comprehensive effects of environmental factors and genetic factors. The environmental factors consist of some harmful substances including ionizing radiation, benzene, mercury, and other pernicious elements. Moreover, long-term hair color, virus infection and long-term use of antibiotics are also harmful and contribute to the occurrence of leukemia.

Apart from these non-genetic risk factors, genetic factors play a vital role in pathogenesis of leukemia. Compared with African-American women, the leukemia morbidity of Latinos and whites is 4–5 times greater. Moreover, the white race suffers from a higher morbidity than the black race and Spanish characters. These results indicate that genetic factors are crucial for leukemia pathogenesis. As far as we know, this is the first meta-analysis which comprehensively explores the association between XRCC3 Thr241Met polymorphism and leukemia susceptibility. It should be noted that Yan et al. published a literature in 2014 that also investigates the association between XRCC3 Thr241Met polymorphism and leukemia risk [11]. Regrettably, only seven studies were included in their meta-analysis. They concluded that XRCC3 Thr241Met polymorphism was not associated with leukemia risk. Qin et al. published a literature in 2013 that also investigates the association between XRCC3 Thr241Met polymorphism and leukemia risk [12]. Similarly, they also get a negative result. Compared with the previous meta-analysis, some important advantages of our paper should be pointed out. Firstly, more eligible studies were enrolled in our meta-analysis. By this means, 16 literatures (10 Caucasian, 4 Asian, and 2 African) were included. Compared with previous meta-analysis, the number of eligible literatures greatly increased. And the merit of meta-analysis is just improving statistical efficiency and making the results more truthful. What’s more, the present study reverses the previous results. We have first discovered that XRCC3 Thr241Met polymorphism contributes an increased risk to leukemia of Caucasian population. The results of our study indicate the limited sample size of previous meta-analysis. So that we think the present meta-analysis is reliable and comprehensive.

We found that XRCC3 Thr241Met polymorphism contributes no risk to leukemia of African and Asian population but contributes an increased risk to leukemia of Caucasian population. To be specific, the T allele and TT genotype were risk factors and they contribute an increased risk to leukemia in Caucasian population. For the past few years, N4-acetylcytidine (ac4C) has been subject to widespread attention as comprehensive modifications have been detected in mRNAs of human and yeast [43]. It contributes to accurately reading codons in the process of translation and improving translational efficiency [43]. Furthermore, there is a direct correlation between ac4C and occurrence, development, progression of number diseases [43].

Eliminating the source of bias is of vital importance for gene polymorphism association meta-analysis. Hence, we have attempted to conduct all the three patterns in the present meta-analysis. Firstly, allele contrast was used to find out the high risk or low risk allele. Secondly, homozygote comparison was used to find out the high risk or low risk genotype. The last pattern is comparing homozygote genotype versus allele carriers. In the present study, the moderate-significant heterogeneity between studies occurred in the overall population. Common reasons for heterogeneity consist of differences in the investigated populations or in genotyping methods or in sample size or it may be derived from other risk factors. By performing meta-regression, and subgroup analysis, we found that ethnicity might contribute substantial heterogeneity to final results. By Galbraith plot analysis, we found the studies Liu et al. and Hamdy et al. were not within reasonable limits. Then we explored the two studies carefully and discovered their shortcomings. The P value < 0.05 of HWE in control group was found in literature of Liu et al. And we found the sample size < 100 participants in literature of Hamdy et al. The results of our meta-analysis were not altered by omitting the two studies. The results of sensitive analysis and publication bias demonstrated that the results of our meta-analysis were stable and reliable.

Although the present meta-analysis is comprehensive and rigorous, there are still some disadvantages existing. Firstly, more studies with different ethnicities are also needed because different ethnicities have different genetic backgrounds. Various ethnicities should be investigated and discussed including African population, Asian population, mixed population and Caucasian population. Secondly, different kinds of confounding factors such as age, gender and radiation exposure are not taken into consideration due to limited dataset [44, 45]. Therefore, more studies in the future on XRCC3 gene considering all of these factors should be performed for subgroup analysis [46, 47]. Thirdly, the relevant GWAS has not been investigated. Thus, rigorous GWAS should be performed for further trans-ethnic and trans-trait meta-analysis [48]. Lastly, if many independent SNPs in other genome regions, XRCC3 Thr241Met polymorphism and environmental factors can be precisely explored, maybe we can establish a machine-learning prediction model, which contributes to early diagnosis for multiple diseases [49, 50].

## Conclusions

The present meta-analysis suggests that the XRCC3 is a candidate gene for leukemia susceptibility. The XRCC3 Thr241Met polymorphism may be risk factor for leukemia in Caucasian population. Further studies investigating other confirmed genetic factors and possible gene–gene and gene-environmental interactions for XRCC3 Thr241Met polymorphism should be performed.

## Supplementary Information

**Additional file 1: Table S1**. PRISMA 2009 checklist

**Additional file 2: Table S2**. PRISMA 2009 Flow Diagram.

**Additional file 3: Figure S3**. Results of evaluating publication bias of other four genetic models (A: homozygote comparison, B: heterozygous comparison, C: recessive genetic model, D: dominate genetic model).

## Data Availability

The datasets used and/or analyzed during the current study are available from the corresponding author on reasonable request.

## Abbreviations

PB: Population-based
HWE: Hardy–Weinberg equilibrium
RFLP: Restricted fragment length polymorphism
NOS: Newcastle–Ottawa Score
OR: Odds ratio
95% CI: 95% Confidence interval
