# Supplementary material for: Ethnicity-stratified analysis of the association between XRCC3 Thr241Met polymorphism and leukemia: an updated meta-analysis
Source: BMC Med Genomics. 2021 Sep 18;14:229. doi: 10.1186/s12920-021-01076-w (PMC8449464; doi:10.1186/s12920-021-01076-w)
Supplement: Supplementary file 2 — Additional file 2: Table S2. PRISMA 2009 Flow Diagram. [file 12920_2021_1076_MOESM2_ESM.doc]

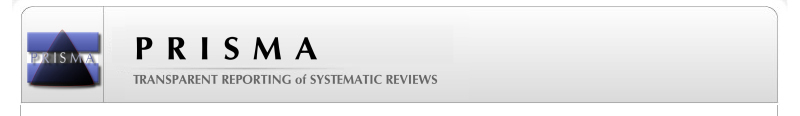
**PRISMA 2009 Flow Diagram**

**Screening**

**Included**

**Eligibility**

**Identification**

Records identified through database searching
(n = 31 )

Additional records identified through other sources
(n = 0 )

Records after duplicates removed
(n =31 )

Records screened
(n =25 )

Records excluded
(n = 4 )

Full-text articles assessed for eligibility
(n = 21 )

Full-text articles excluded, with reasons
(n = 5 )

Studies included in qualitative synthesis
(n = 16 )

Studies included in quantitative synthesis (meta-analysis)
(n = 16 )
