# Supplementary figures and images for: Ethnicity-stratified analysis of the association between XRCC3 Thr241Met polymorphism and leukemia: an updated meta-analysis
Source: BMC Med Genomics. 2021 Sep 18;14:229. doi: 10.1186/s12920-021-01076-w (PMC8449464; doi:10.1186/s12920-021-01076-w)

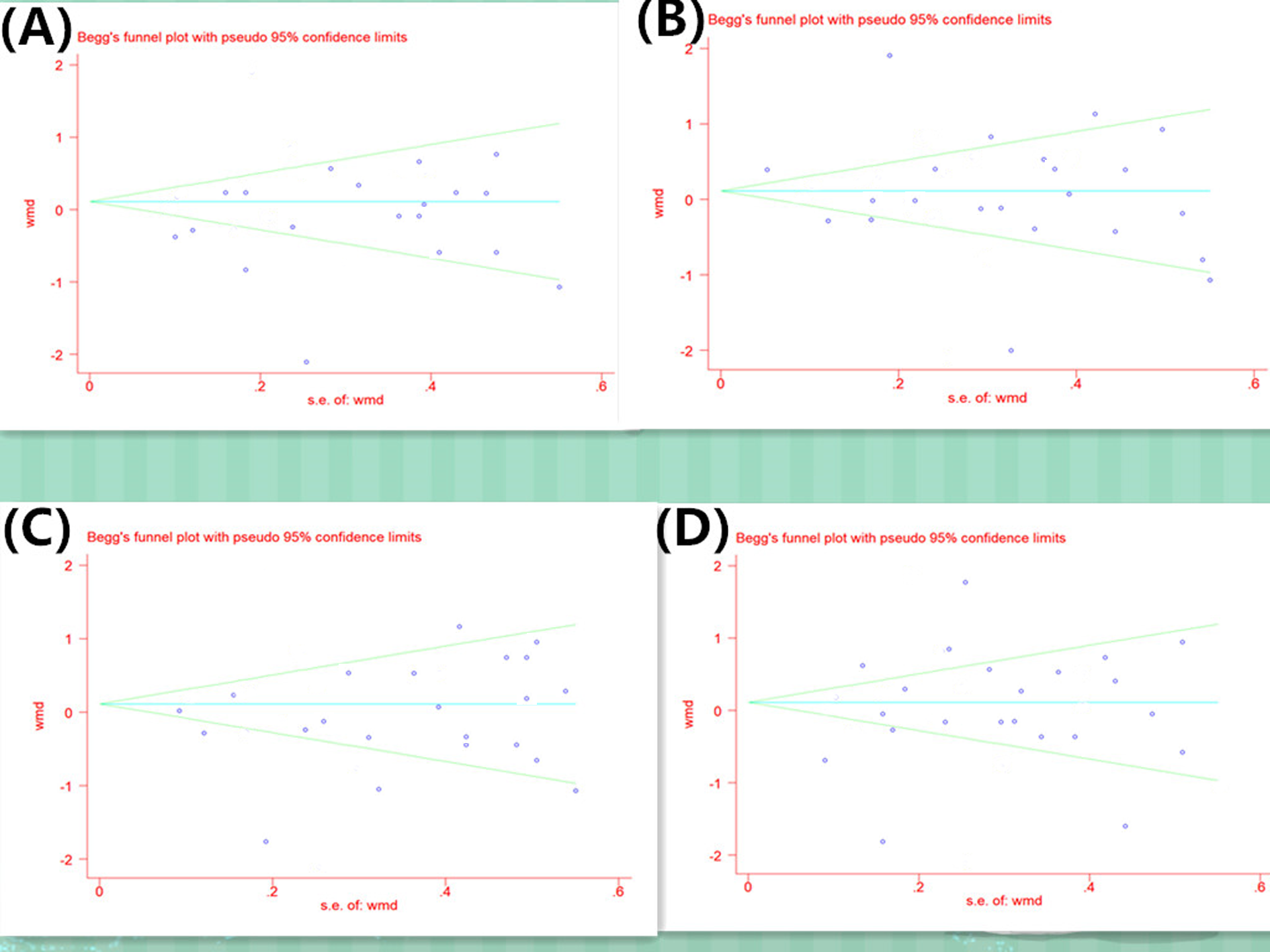

Supplement: Supplementary file 3 — Additional file 3: Figure S3. Results of evaluating publication bias of other four genetic models (A: homozygote comparison, B: heterozygous comparison, C: recessive genetic model, D: dominate genetic model). [file 12920_2021_1076_MOESM3_ESM.tif]
